# Microencapsulation of Beetroot Anthocyanins: Investigation of Degradation Kinetics and Modeling by Using Artificial Neural Networks

**DOI:** 10.1021/acsomega.5c12228

**Published:** 2025-12-25

**Authors:** Tugca Bilenler Koc, Ilkay Fırat, Ihsan Karabulut, Cihangir Boztepe, Zeynal Topalcengiz

**Affiliations:** † Department of Food Engineering, Faculty of Engineering, 37520 Inonu University, Malatya 44280, Turkey; ‡ Department of Biomedical Engineering, Faculty of Engineering, Inonu University, Malatya 44280, Turkiye; § Department of Food Engineering, Faculty of Engineering and Architecture, Muş Alparslan University, Mus 49250, Turkiye; ∥ Department of Food Science, Center for Food Safety, University of Arkansas System Division of Agriculture, Fayetteville 72704, Arkansas, United States

## Abstract

Anthocyanins are widely appreciated as natural pigments, but their use in foods and related industries is still quite limited because they are highly sensitive to heat, pH changes, light, and oxygen. Improving their stability has therefore become a key focus in developing more reliable natural color systems. In this study, beetroot anthocyanins were microencapsulated with different wall materials, maltodextrin (MD), gum arabic (GA), a simple MD/GA blend, and a ternary structure combining MD, GA, and sodium caseinate (MD/GA/SC). These systems were evaluated for their encapsulation efficiencies, antioxidant activity preservation, release behaviors, and degradation responses over a wide range of temperatures (40–100 °C) and pH levels (2.5–6.5). Remarkable findings demonstrated that the MD/GA/SC formulation provided the highest encapsulation efficiency (93.36%), superior radical-scavenging activity (88.43%), and the most controlled release profile. Moreover, this formulation demonstrated the lowest degradation rate constants at pH 2.5, 4.5, and 6.5 (2.886, 2.083, and 1.30 1/min, respectively) together with the highest activation energies at these pH levels (37.460, 52.517, and 62.045 kJ/mol, respectively), indicating a pronounced improvement in thermal stability compared with the other formulations and the free extract. An artificial neural network (ANN) model was developed to predict anthocyanin degradation. The ANN provided highly accurate predictions (*R* ^2^ > 0.98, RMSE < 0.01) across all conditions and outperformed the classical first-order kinetic model. These findings highlight the potential of the MD/GA/SC matrix as a promising encapsulation system for improving anthocyanin stability. The strong performance of the ANN model also suggests that data-driven approaches can contribute meaningfully to designing more reliable microencapsulation strategies for future food and nutraceutical applications.

## Graphical abstract

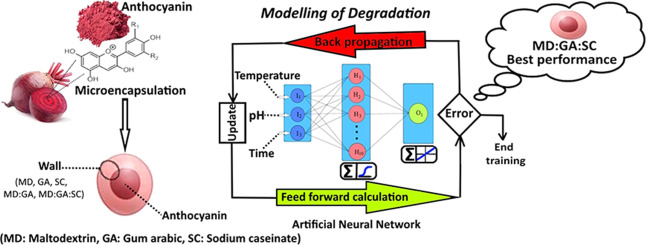

## 1 Introduction

Anthocyanins are structurally classified in the flavonoid group of water-soluble phenolic compounds that are responsible for the characteristic red, blue, purple, and orange colors of many fruits and flowers.^1,2^ To date, more than 600 anthocyanins have been identified. Anthocyanin derived from flavonol has a keto oxygen-free flavylium ion in the 4-position with an empirical formula of C_15_H_11_O_+_ and a molecular weight of 207.24724 g/mol. Anthocyanins are defined depending on the location and number of hydroxyl groups, the degree of methylation of the hydroxyl groups, the number and nature of the sugar (glycone) molecules attached to the aglycone part, and the degree of acylation degree of the sugar (aliphatic or aromatic acid) molecules.^3,4^ Nontoxicity, striking colors, and high water solubility properties offer the use of anthocyanins as natural coloring agents.^1,5^ Anthocyanins are also known for various health benefits as strong antioxidant activity, anticancer, antidiabetic agent, and antimutagenic properties.^6^ However, high polarity of anthocyanins and pH changes during the gastrointestinal passage and instabilities caused by microbial degradation may result in low in vivo absorption of anthocyanins. The pharmacokinetic properties (bioavailability, metabolism, and excretion) of anthocyanins affect their biological activities dramatically.^7^

Anthocyanins have gained considerable attention in the food and nutraceutical fields, not only because they provide attractive natural color but also due to their notable antioxidant properties. Despite this growing interest, their practical use is still quite restricted, mainly because these pigments tend to degrade easily during processing or storage. For this reason, improving their stability has become an essential step for maintaining product quality, extending shelf life, and enabling wider industrial applications. In the past few years, various microencapsulation and delivery approaches have been explored as ways to protect sensitive bioactive molecules, including polyphenols, essential oils, carotenoids, and plant-derived extracts, by improving their stability, bioaccessibility, and release control^1,6^ . Different types of biopolymer-based coatings, protein-polysaccharide mixtures, and even nanoemulsion-assisted systems have shown encouraging outcomes in prolonging the functional behavior of compounds that are otherwise prone to rapid degradation. For example, coatings made from gelatin and aloe vera, when combined with nanoemulsions of Shirazi thyme essential oil, were found to noticeably extend the freshness and overall quality of button mushrooms.^8^ Likewise, incorporating microencapsulated propolis extract into yogurt helped maintain its functional attributes throughout storage.^9^ Another recent study demonstrated that encapsulated pummelo essential oil provided strong protection and improved stability, contributing to a better shelf life in *Agaricus bisporus* mushrooms.^10^ Together, these findings underline how important encapsulation technologies have become for safeguarding fragile bioactives and enhancing the stability of real food products.

Encapsulation can be defined as a process to entrap bioactive agents into a coating material.^1^ The most common practices for microencapsulation include freeze-drying or spray drying of the emulsion formed by homogenizing the core and wall material mixture. Different wall materials, such as carbohydrates, proteins, gums, and fibers, can be used alone or in combination during the encapsulation process^11^. Maltodextrin is the most widely used wall material due to its low cost. In addition, gums are combined with maltodextrin to increase encapsulation efficiency.^12^ Maltodextrin alone,^13^ a mixture of maltodextrin and xanthan gum,^7^ soy protein alone,^14^ and inulin^15^ have been used in the encapsulation of beetroot extract with high anthocyanin content.

Recent studies in this area have shown noticeable progress, especially with the use of biopolymer-based carriers, mixed protein-polysaccharide systems, and various nanoemulsion-supported protective techniques. Some of these encapsulation strategies have even been applied directly in real food systems, such as mushrooms, yogurts, fruit beverages, and other functional drinks, which demonstrates how far the field has come. Even with these promising developments, there are still a number of issues with which researchers continue to deal with. For instance, finding the most suitable combinations of wall materials that can reliably enhance the stability under different processing conditions remains a key challenge. Similarly, achieving a predictable and truly controlled release in complex food matrices is not always straightforward. Another issue is the difficulty in forecasting degradation behavior, since the breakdown of sensitive compounds often depends on several environmental factors that interact in nonlinear ways.^16,17^ Although microencapsulation is widely explored as a strategy to stabilize anthocyanins, there is still a need to systematically compare different wall material combinations and identify formulations that provide superior thermal and pH stability. In particular, the stabilizing potential of a ternary maltodextrin-gum arabic-sodium caseinate (MD/GA/SC) matrix has not been comprehensively evaluated, creating a clear gap in the current literature. Therefore, this study aims to systematically evaluate the microencapsulation of beetroot anthocyanins using maltodextrin (MD), gum arabic (GA), their binary blend (MD/GA), and a ternary maltodextrin-gum arabic-sodium caseinate (MD/GA/SC) system and to investigate their stability under varying pH and temperature conditions.

The anthocyanin release rate and degradation process are influenced by several factors including material type, coating material-substrate interactions, pore closure-relaxation, pore structure, pH, temperature, light, oxygen, enzymes, metal ions and solvent types.^17^ The accurate prediction of anthocyanin degradation behavior is difficult due to the numerous parameters mentioned above. Mathematical modeling developed for the degradation behavior of anthocyanin is as important as the design of high-performance anthocyanin coating materials. The characterization of the degradation kinetics of anthocyanin extracts has generally been studied with first-order kinetic model or Weibull model^18^ but there is no current artificial intelligence-based model that can modeling the complex degradation processes in the degradation kinetics of free and microencapsulated anthocyanin with high coherence. Despite recent advances in encapsulation technologies, several challenges persist in effectively stabilizing anthocyanins under diverse processing conditions. Identifying optimal combinations of wall materials remains a major research need, particularly for complex ternary systems, such as maltodextrin (MD), gum arabic (GA), and sodium caseinate (SC), whose synergistic effects on anthocyanin stability have not been fully elucidated. Moreover, predicting anthocyanin degradation is inherently difficult, as the process is governed by multiple interacting factors, including temperature, pH, oxygen exposure, and the molecular microenvironment, that collectively influence pigment integrity. This multifactorial and nonlinear degradation behavior cannot be accurately captured using traditional first-order kinetic models, which are often limited in representing the multivariate nature of anthocyanin breakdown. These limitations underscore the necessity of employing advanced data-driven predictive tools capable of modeling complex degradation pathways under varying pH and temperature conditions. The artificial neural network (ANN) is one of the artificial intelligence techniques based on the working principle of the human brain. ANN acquiring new information through learning create new information and realization about their ability for faster and cheaper solutions for problems in many fields, especially engineering and food safety.^19,20^ The ANN technique has been successfully used in many food processes including extraction, extrusion, drying, filtration, canning, fermentation, baking, dairy processing, and quality evaluation to modeling with high performance and excellent prediction capability.^21^ The main function of an ANN is to perform machine learning by enabling the computer to learn. To train the network, examples must be defined and learned by using the data. Thus, when they encounter similar events, they can learn about events and make logical decisions, as well as produce information about examples that have not been seen before.^22^ No studies to date have applied artificial neural network (ANN) modeling to predict the degradation of microencapsulated anthocyanins. This represents a significant research gap, as ANN provides a powerful data-driven framework capable of handling nonlinear interactions and offering higher predictive accuracy. Addressing this gap is important for advancing predictive modeling in anthocyanin stabilization and for developing more intelligent and optimized encapsulation systems.

Beetroot (*Beta vulgaris L.*) is rich in compounds with nutritional value and biological functions.^21^ Betalains, carotenoids, and anthocyanins, responsible for the color of beetroots, are used as natural coloring agents in the food industry.^22,23^ However, the use of anthocyanins in the food industry is limited due to their high sensitivity to factors such as pH, temperature, light, oxygen, enzymes, metal ions, and solvent type during food processing and storage.^1,11^ In this context, this study aims to identify the most effective wall material for microencapsulating beetroot anthocyanins and to evaluate their stability under varying pH and temperature conditions representative of food processing environments. Beetroot extract rich in anthocyanins was encapsulated using maltodextrin (MD), gum arabic (GA), and sodium caseinate (SC), applied individually or in combination via freeze-drying. This study introduces several innovations that have not been comprehensively addressed in previous research. Unlike previous studies that mostly focused on single wall materials or limited environmental conditions, this study provides a systematic comparison of MD, GA, MD/GA, and the novel MD/GA/SC ternary matrix across a wide pH and temperature range. The degradation behavior of microencapsulated anthocyanins across different pH and temperature conditions was kinetically examined and further modeled by using an artificial neural network (ANN). Integrating ANN analysis provides a novel data-driven prediction approach that addresses the limitations of conventional kinetic models. By identifying an optimal encapsulation matrix and demonstrating the predictive capability of ANN-based modeling, this study offers a meaningful advancement toward the development of more stable and robust anthocyanin delivery systems for food and functional applications.^24^

## 2 Materials and Methods

### 2.1 Chemicals

Maltodextrin (16.5–19.5 DE), gum arabic, sodium caseinate, 2,2-diphenyl-1-picrylhydrazyl (DPPH), α- amylase from human saliva (A1031), pepsin from porcine gastric mucosa (P7125), bile salt bovine (B3883), and phosphate buffer solution were purchased from Sigma-Aldrich (St. Louis, MO, USA). Pancreatin (A0585) was purchased from AppliChem (Darmstadt, Germany). Methanol, HCL, and NaOH were purchased from Merck KGaA (Darmstadt, Germany). Potassium chloride buffer and sodium acetate buffer solutions were obtained from Norateks Kimya (İstanbul, Türkiye).

### 2.2 Beetroot Extraction

Beetroots were purchased from a local grocer in Malatya (Türkiye). After being washed with running tap water, beetroots were peeled with a clean knife. Deionized water was added to the grated beetroot at a ratio of 1:1 and slurried by using T18 Ultra Turrax (Ika Works, Inc., Staufen, Germany) at 10,000 rpm for 5 min. The upper aqueous phase was separated by centrifugation (Nüve, NF 400, Ankara, Türkiye) at 4500 rpm for 15 min. The supernatant was filtered and frozen at −18 °C overnight. The beetroot extract was dried in a freeze-dryer (Lyovapor L-200, Buchi, Sweden) at 0.200 mbar for 20 h and stored in airtight containers at −18 °C until use^25^.

### 2.3 Encapsulation Process

The microencapsulation of beetroot extract was performed using the freeze-drying (lyophilization) method, following the protocol proposed by Bazaria and Kumar^25^ with minor modifications. Initially, the freeze-dried beetroot extract powder was dissolved in deionized water to obtain a solution with a total soluble solid content of 10 °Bx. Wall materials, maltodextrin (MD), gum arabic (GA), and sodium caseinate (SC), were separately dispersed in deionized water under constant magnetic stirring until complete hydration was achieved, according to the formulation ratios (F1, F2, F3, and F4) given in Table 1. The hydrated wall material solutions were mixed on a magnetic stirrer at 500 rpm for 24 h at room temperature to ensure the full homogenization of the encapsulating matrix. Subsequently, the beetroot extract solution was slowly added to the wall material mixture and stirred at 500 rpm for an additional 30 min to facilitate the uniform incorporation of the core material. The resulting emulsified mixture was then subjected to freeze-drying: samples were frozen at −40 °C and lyophilized under vacuum conditions (0.01–0.02 mbar) until the complete removal of moisture. The obtained microencapsulated powders were collected, sealed in airtight containers, and stored at −18 °C in the absence of light until further analysis.

**Table 1:** Formulations of Wall and Core Material^a^

formulation number	wall materials (g/100 gof solution)			core material (g/100 g of solution)
	MD	GA	SC	beetroot extract
F1	5	-	-	10
F2	-	5	-	10
F3	2.5	2.5	-	10
F4	1.87	1.87	1.25	10

a MD: Maltodextrin, GA: Gum arabic, SC: Sodium caseinate.

### 2.4 Total Anthocyanin Content Analysis

Total anthocyanin content was determined by using the spectrophotometric pH differential method.^27^ The samples were diluted separately with 0.25 M potassium chloride buffer (pH 1) and 0.4 M sodium acetate buffer (pH 4.5). The absorbance of the mixtures was measured at 520 and 700 nm by using a UV–vis spectrophotometer (Shimadzu, Kyoto, Japan). The absorbance was determined with eq 1.

1 A=[(A520−A700)pH1.0−(A520−A700)pH4.5]

The total anthocyanin content was calculated as the cyanide 3-glucoside equivalent by using the following eq 2.

2 $$Anthocyanin\;content\;(\frac{mg}{100}g=\frac{A\times M_{W}\times DF\times V\times 100}{\varepsilon \times I\times xm_{sample}}$$

where *A* is the absorbance, *M*_W_ is the molecular weight (*M*_W_ = 449.2 g/mol), *DF* is the dilution factor, ε is the molar absorptivity (ε = 26900 L/cm mol), *V* is the volume of solvent in mL, *l* is the path length (cm), and *m*_sample_ is the sample amount (g).

### 2.5 Encapsulation Efficiency

Total anthocyanin (TA) and surface anthocyanin (SA) amounts were determined to calculate the encapsulation efficiency. To determine the total amount of anthocyanin, 100 mg of microcapsules were vortexed with 1 mL of distilled water in a screw-capped tube for 2 min. Nine mL of ethanol was added and filtered after 5 min to extract the anthocyanins. To determine the amount of surface anthocyanin, 100 mg of microcapsule was added to 10 mL of ethanol and vortexed for 30 s. The mixture was centrifuged (Nüve, NF400, Ankara, Türkiye) at 3000 rpm for 10 min, and the supernatant was collected for filtration. The total anthocyanin and surface anthocyanin amounts were determined by using the pH differential method, as described above. The encapsulation efficiency was calculated with the following eq 3.^26^

3 $$\%\;Encapsulation\;efficiency=\frac{\left(TA-SA\right)}{TA}\times 100$$

where: TA: total anthocyanin, SA: surface anthocyanin.

### 2.6 Morphology

The morphology of the microcapsules was investigated by scanning electron microscopy (SEM; Leo EVO-40 VPX, Carl Zeiss SMT, Cambridge, UK) after coating with a gold–palladium mixture under a vacuum.

### 2.7 Radical Scavenging Activity

The radical scavenging activity of the microencapsulated beetroot extract was determined by the DPPH scavenging capacity of free beetroot extract and empty (blind) microcapsules. Free beetroot extract (5 mg), microcapsules containing an equivalent amount of beetroot extract (calculated according to the results of encapsulation efficiency), and empty microcapsules were solved and weighed into screw-capped glass tubes. DPPH radical solution prepared in 80% methanol was added (10 mL) into tubes. The radical scavenging test was monitored for 180 min, and samples were withdrawn at periodic intervals (every 15 min). The absorbance of the samples was measured at 520 nm using a UV-1700 Spectrophotometer (Shimadzu, Kyoto, Japan). The percent inhibition of the samples was calculated with the following eq 4

4 $$\%\;inhibition=\frac{A_{B-A_{A}}}{A_{B}}\times 100$$

where: *A*_B_—absorbance of blank sample (*t* = 0 min); *A*_A_—absorbance of sample.

Radical scavenging activity analysis was performed in triplicate, and the results were given as mean ± standard deviation.

### 2.8 Static In Vitro Digestion

A static model was used to simulate the digestive conditions in the mouth, stomach, and intestines for the determination of release profile of the microencapsulated beetroot extract.^27^ The simulated saliva solution was prepared by dissolving 0.2% α-amylase in phosphate buffer (pH 6.8 ± 0.2). The simulated gastric juice (SGJ) was prepared by dissolving pepsin (3 g/L) in a sterile NaCl solution (9 g/L) with an adjusted pH of 3 by adding 1.0 mol/L HCl. Bile salt (3 g/L) and pancreatin (10 g/L) were dissolved in phosphate buffer by adjusting to pH 8 with 0.1 M NaOH to prepare simulated intestinal fluid (SIJ). The digestion experiment was carried out by using 100 g of sample in a capped bottle at 37 °C with magnetic stirring (500 rpm). The samples were sequentially released into the digestion media. In the first step, 10 mL of saliva was added to mimic an oral medium; 1 mL of sample was taken after mixing for 5 min. In the second step, 10 mL of SGJ was added to simulate the stomach medium for sampling (1 mL) every 30 min for 1 h. Finally, 10 mL of SIJ was added to create the intestinal environment, and 1 mL of sample was taken at 2 and 4 h. After centrifugation at 4500 rpm for 5 min, the samples were filtered through a 0.22 μm membrane filter (MF-Millipore, USA). The total released anthocyanin content was determined by using the pH differential method described in the previous sections^27^. Experiments were performed in triplicate (*n* = 3).

### 2.9 Thermal Degradation

The success of the wall material formulations given in Table 1 in preserving beetroot anthocyanin at different pH values (2.5, 4.5, and 6.5) and temperatures (40, 60, 80, and 100 °C) was determined by measuring the total anthocyanin content at regular time intervals (0, 30, 60, 120, 240, and 360 min)^27^. Phosphate buffer solutions used in the test were prepared with 0.1 N HCl and 0.1 N NaOH. A total of 30 mg of beetroot extract and microcapsules containing an equivalent amount of beetroot extract were added to phosphate buffer solutions (10 mL) adjusted to different pH levels separately in screw cap tubes at each time interval. Experiments were carried out by immersing test tubes in a water bath to the test temperatures mentioned above. The samples in the water bath was taken at each time point and placed onto the ice bath for quick cooling. After filtration, the total anthocyanin content was determined as described above. Experiments were performed in triplicate (*n* = 3).

### 2.10 Kinetic Data on Anthocyanin Degradation

The anthocyanin concentration changes of beetroot extract in microcapsules during thermal treatment was evaluated by using kinetic parameters. The reaction rate expression for the degradation kinetics, as applied by Boekel,^28^ is as follows eq 5

5 $$-\frac{d\left[C\right]}{dt}=k{\left[C\right]}^{m}$$

where *C* is the anthocyanin concentration, *k* is the reaction rate constant, and m is the order of the reaction.

As reported by Eyarkai Nambi et al., zero order (6) and first-order (7) models^25^ can be derived as follows

6 $$C=C_{0}+kt$$

7 $$C=C_{0}\exp\left(-kt\right)$$

where *C*_0_ is the initial concentration of anthocyanin, *k* is the reaction rate constant, and *t* is the time.

The half-life time (*t*_1/2_) is the time required for degradation of the anthocyanin content. The half-life time of the first-order kinetics reaction was calculated using eq 8

8 $$t_{1/2}=\frac{\left(-0.693\right)}{k}$$

The effect of temperature on anthocyanin degradation was defined by the Arrhenius eq 9 and expressed as activation energy (*E*_a_)^29^

9 $${k=k}_{0}\;\exp\left(\frac{E_{a}}{RT}\right)$$

where *k*_0_ is the Arrhenius constant, *R* is the universal gas constant (8.3145 J/mol K), *T* is the absolute temperature (*K*), and *E*_a_ is the activation energy (kJ/mol)

### 2.11 Design of Artificial Neural Network Model

An Artificial neural network (ANN) model was trained to model the degradation behavior of anthocyanins in free and microencapsulated beetroot. This model utilized experimental data as an input set to identify the effects of time, pH, and temperature on the degradation of anthocyanins. All ANN modeling procedures were performed using MATLAB (MathWorks, USA) with the Levenberg–Marquardt back-propagation algorithm as the training function. The use of a fixed seed, multiple training repetitions, and a clearly defined neuron selection strategy enhance the transparency and reproducibility of the ANN methodology applied in this study. A three-layer feed-forward back-propagation neural network with logarithmic sigmoid transfer function (logsig) at a hidden layer and a linear transfer function (purelin) at an output layer were chosen. Generalized ANN topology to predict the anthocyanin degradation of free and microencapsulated beetroot was illustrated in Figure 1, showing the connections between inputs and the artificial neurons. The input data with a 360-point data set included three data sets in total: temperature (°C), pH, and time (*h*) in the input layer. The ANN model utilized these experimental data sets to identify the anthocyanin concentration remaining in the structure of beetroot extract. The selection of the number of neurons in the hidden layer was carried out through a structured trial-and-error optimization procedure. Various architectures containing 3 to 15 neurons were systematically tested. For each configuration, model performance was evaluated based on validation *R* ^2^ and RMSE. The final architecture, comprising one hidden layer with 10 logarithmic sigmoid (logsig) functions, was chosen because it produced the highest validation *R* ^2^ and the lowest RMSE while avoiding signs of overfitting, such as divergence between training and validation errors. This systematic tuning ensured a balance between the model complexity and generalization performance. The momentum coefficient was used as an assigned default value of 0.9 by the program. The transfer functions called “logsig” in Matlab is given as follows eq 10.

10 $$y_{i}=\frac{1}{1+e^{-z_{i}}}-1$$

where *z*_*i*_ is the input of the neuron in the hidden layer and *y*_*i*_ is the output of the neuron while calculating. The log sig transfer function calculated a layer’s output from its net input. Data sets were divided into two subsets randomly. In this model, 70 percent of the data was used for training, while the remaining was used for testing. For these models, the Levenberg–Marquardt method was used for optimization. The developed ANN models were tested with test data that were not used during training. The success of the model was measured using statistical techniques. The optimal architecture of the ANN model and its parameter variations were determined based on the minimum value of the MSE of the training and prediction set. The data set was divided into training (70%), validation (15%), and testing (15%) sets. The network was trained for a maximum of 5000 epochs. Matlab and the neural network toolbox were used in the development of the ANN model.

11 $$R^{2}={\left(\frac{\sum_{m=1}^{N}\left(x_{m}-\mathrm{x̅}\right)\left(y_{m}-\mathrm{y̅}\right)}{\sqrt{\sum_{m=1}^{N}{\left(x_{m}-\mathrm{x̅}\right)}^{2}}\sqrt{{\sum_{m=1}^{N}\left(y_{m}-\mathrm{y̅}\right)}^{2}}}\right)}^{2}$$

12 $$RMSE=\sqrt{\frac{\sum_{m=1}^{N}{\left(y_{m}-x_{m}\right)}^{2}}{N}}$$

13 $$MSE=\frac{1}{N}\sum_{m=1}^{N}{\left(y_{m}-x_{m}\right)}^{2}$$

14 $$MAPE\left(\%\right)=\frac{1}{N}\sum_{m=1}^{N}(|\frac{y_{m}-x_{m}}{x_{m}}|).100$$

where *x*_*m*_ is an observed value at the *i* ^th^ time step, *y*_*m*_ is a simulated value at the same moment of time, *N* is the number of time steps, *x̅* is the mean value of observations, and *y̅* is the mean value of simulations in these equations.

**Figure 1:**
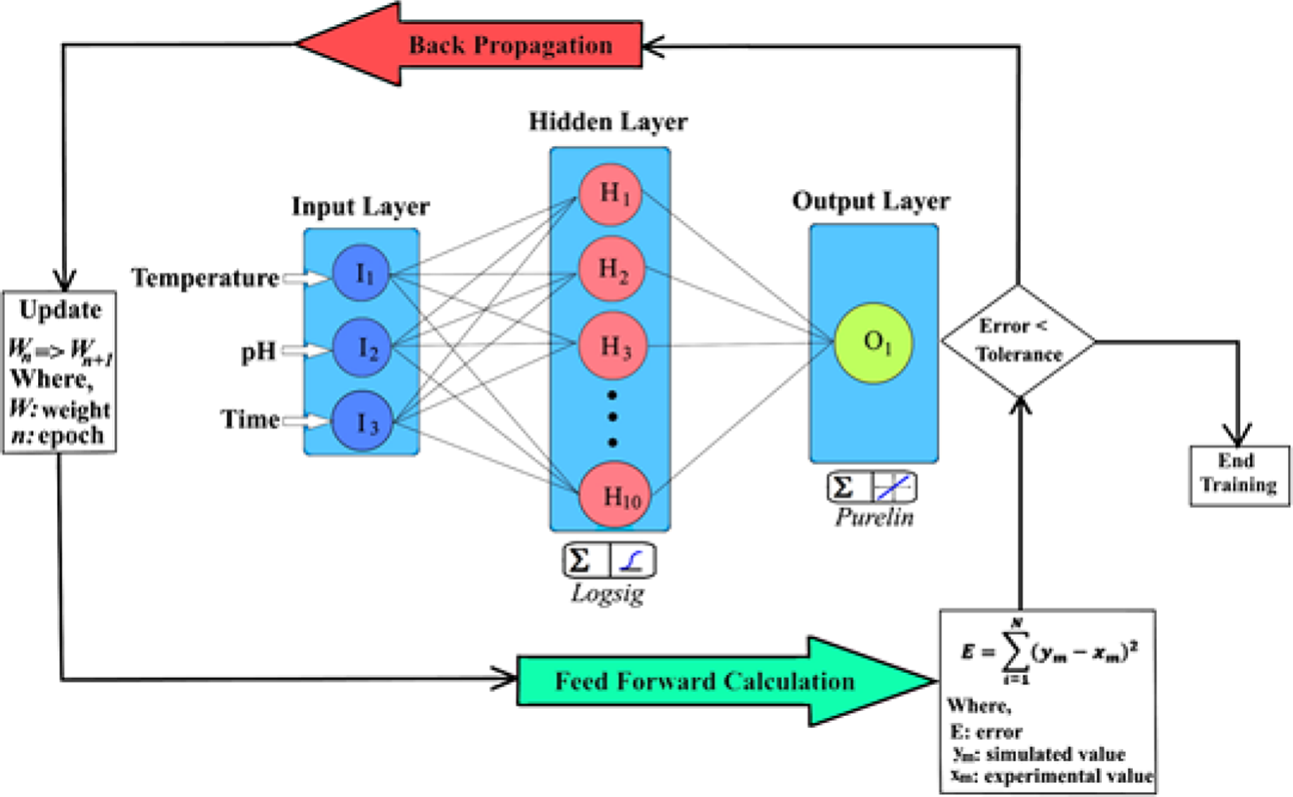
Feed-forward back-propagation ANN topology developed to predict the degradation of the anthocyanin. Several statistical indicators, including the coefficient of determination (*R* ^2^), root-mean-square error (RMSE), mean square error (MSE), and mean absolute percentage error (MAPE) (%) were used to evaluate the performance of constructed networks. *R* ^2^, RMSE, MSE, and MAPE were obtained through the following calculations.

## 3 Results and Discussion

### 3.1 Encapsulation Efficiency

Based on data from previous studies and wall material cost, MD and GA were chosen as the two main wall materials in this study.^12,13,25^ The highest encapsulation efficiency (%) value was determined in the formulation prepared with the MD/GA/SC mixture (95.36 ± 0.18), followed by GA (90.34 ± 0.05), MD/GA (89.60 ± 1.02), and MD (88.54 ± 0.50). The lowest encapsulation efficiency was determined in MD microcapsules due to the low emulsification capacity of MD.^30^ It is common practice to use MD together with GA to eliminate insufficient emulsification capacity. Previously, the encapsulation efficiency was determined as 94.30% when MD was used alone in the encapsulation of lemon essential oil, while the use of MD/GA at a 1:1 ratio increased encapsulation efficiency to 98.95%.^31^ In another study, the encapsulation efficiency values for the encapsulation of propolis phenolics with MD alone and MD/GA mixture (1:1 ratio) were reported to be 14.9% and 49.2%, respectively.^32^

The highest encapsulation efficiency was determined in the MD/GA wall combination supplemented with 25% SC based on preliminary experiments testing 10%, 20%, and 25% SC concentrations (data not shown). None of the wall materials alone meets all of the features that the ideal wall material should have. Research on improving the properties of wall materials has focused on carbohydrate: protein blends.^33^ These results indicate that the presence of sodium caseinate enhances the structural integrity and intermolecular interactions within the encapsulation matrix. Where polysaccharide-protein hybrid structures demonstrated improved emulsification stability and encapsulation performance. In this regard, sodium caseinate draws attention due to its high emulsification ability, not being denatured during drying and thus increasing the encapsulation efficiency.^30^

### 3.2 Morphology

Representative images of empty and beetroot extract loaded microcapsules prepared with MD, GA, MD/GA, and MD/GA/SC wall material formulations are given in Figure 2A–H, respectively. The surface structures of the microcapsules exhibited broken glass and flake-like irregular structures. In many studies, it has been reported that freeze-dried microparticles have a similar morphology and many characteristics as sublimation and glass transition temperature that are effective in the formation of these structural shapes.^11,34,35^

**Figure 2:**
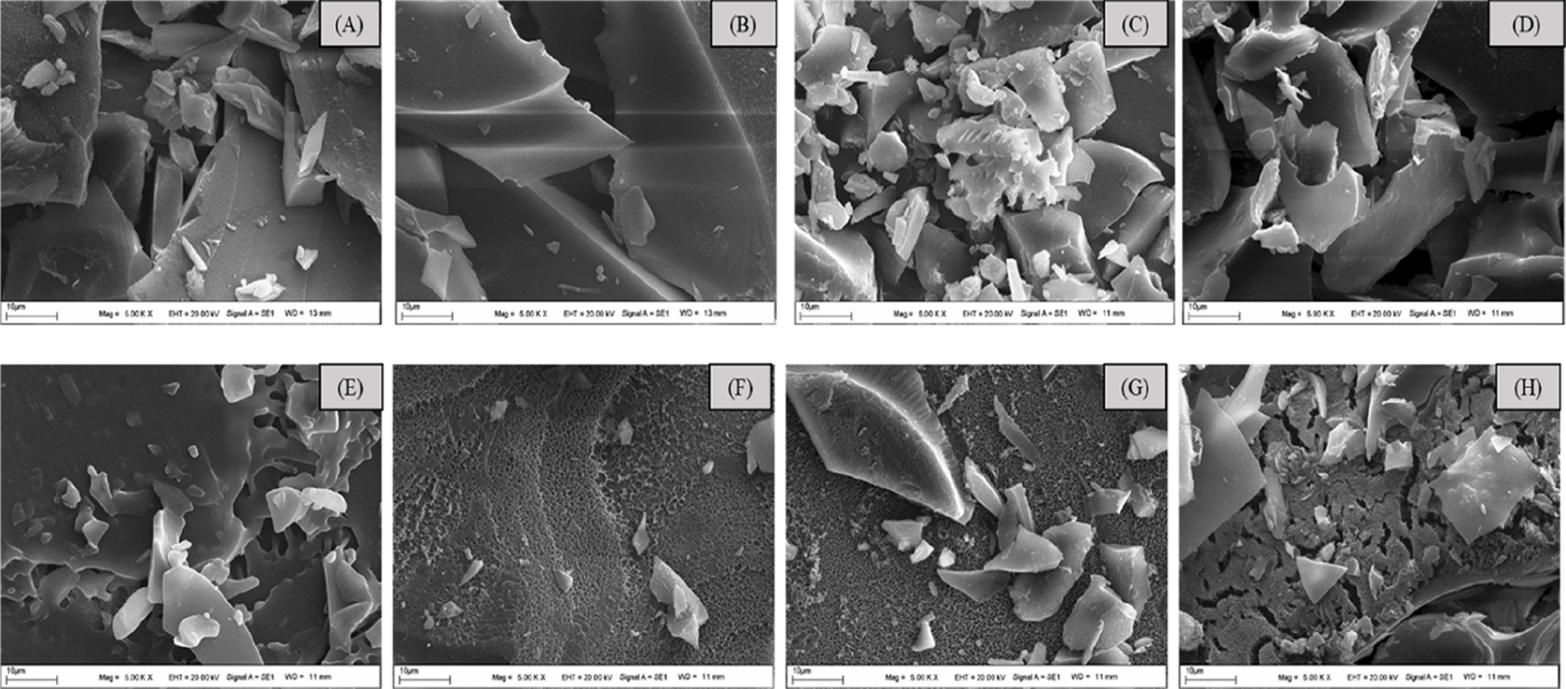
SEM images of microcapsules prepared from different wall material formulations as follows: (A) MD without the beetroot extract; (B) GA without the beetroot extract; (C) MD/GA without the beetroot extract; (D) MD/GA/SC without the beetroot extract; (E) MD with the beetroot extract; (F) GA with the beetroot extract; (G) MD/GA with the beetroot extract; and (H) MD/GA/SC with the beetroot extract.

In addition, the drying method also affects the morphological structure. In the study by Bazaria and Kumar,^25^ the spray-drying technique in the microencapsulation of beetroot extract by using MD and GA as the wall material resulted in spherical microcapsules. In another study, the propolis extract was microencapsulated in MD, GA, and Inulin as wall material combinations with either a freeze-drying or spray-drying technique. The researchers reported that the spray-dried particles exhibited an ideal spherical shape, while the freeze-dried particles exhibited an irregular broken glass structure.^32^

The image of the empty microcapsules (Figure 2A–D) is quite different from that of the microencapsulated beetroot extract (Figure 2E–H), except for the MD formulation. The results clearly show that the presence of beetroot extract in the microcapsule structure caused the formation of porous structures on the surfaces of the GA, MD/GA, and MD/GA/SC formulations. The removal of frozen water from the structure by sublimation during the freeze-drying period causes the formation of a porous surface.^34^ An obvious shrinkage was observed in the MD/GA (Figure 2G) formulation but not in the MD/GA/SC (Figure 2H) formulation. Hee et al.^30^ reported that the shrinkage disadvantage would be eliminated by adding SC to the wall material formulation in microencapsulation.

### 3.3 Radical Scavenging Activity

DPPH radical scavenging activities of free beetroot extract and beetroot extract-loaded MD, GA, MD/GA, and MD/GA/SC microcapsules followed by 180 min are given as percent inhibition in Figure 3A. At the beginning of the test (first 15 min), the radical scavenging powers of free and microencapsulated (MD, GA, MD/GA, and MD/GA/SC) beetroots were 38.91% and 22.65%, 21.92%, 29.10%, and 36.36%, respectively. The decrease in scavenging activities with the microencapsulation process is explained by the strong bonding between the wall and the core material.^36^ Similar results were reported by many researchers. In agreement with our results, García-Segovia et al.^37^ determined that the DPPH cleaning power of beetroot extract decreased from 314 mg Trolox equivalent/100 g beetroot solids to 213–304 mg Trolox equivalent/100 g beetroot solids by microencapsulation with spray drying and pea protein. In another study, the antioxidant activity of beetroot extract (5.70Abs-–3/min/mg dry matter) was higher than those microencapsulated with gum arabic (4.70Abs-–3/min/mg dry matter).^38^ ^,^ ^39^

**Figure 3:**
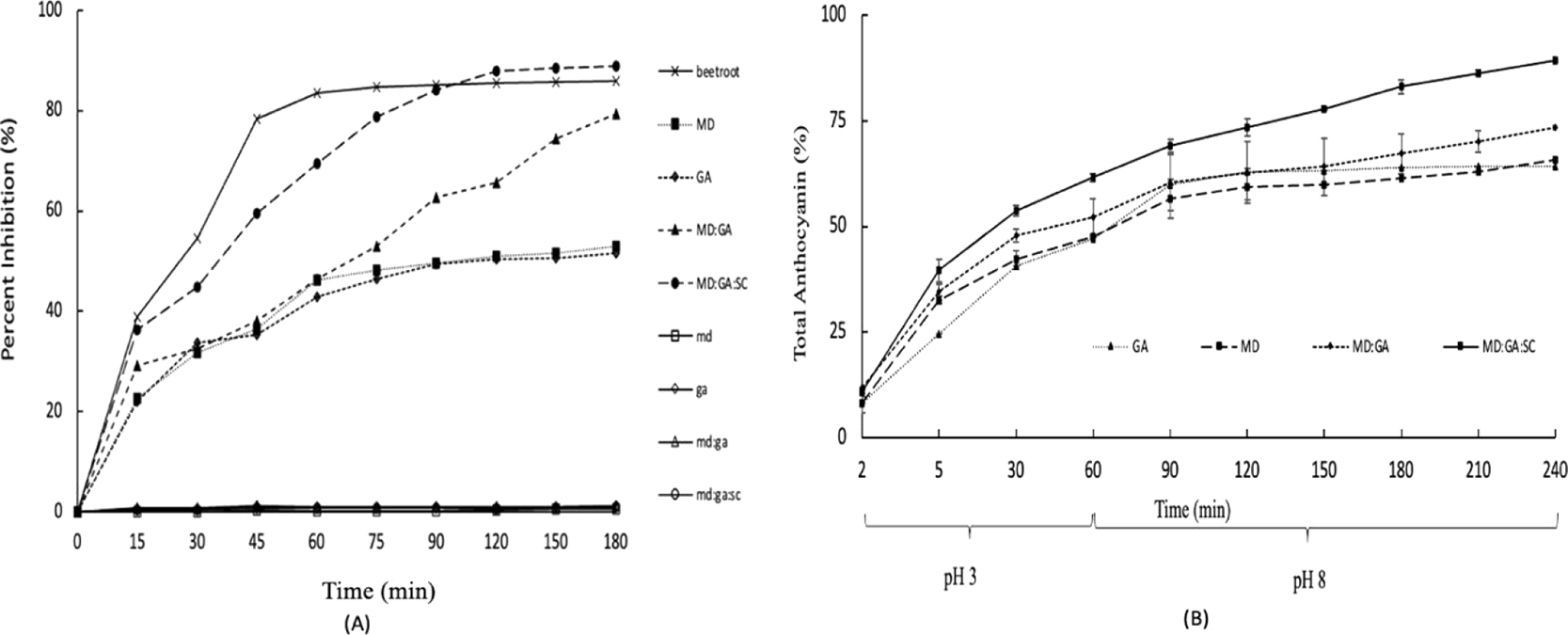
(A) Radical scavenging activity (percent inhibition) of beetroot extract, beetroot extract-loaded maltodextrin (MD), gum arabic (GA), maltodextrin-gum arabic (MD/GA), maltodextrin-gum arabic-sodium caseinate (MD/GA/SC) and empty (without the beetroot extract) maltodextrin (md), gum arabic (ga), maltodextrin/gum arabic (md/ga), and maltodextrin/gum arabic/sodium caseinate (md/ga/sc). (B) The cumulative release of total anthocyanin from beetroot extract-loaded maltodextrin (MD), gum arabic (GA), maltodextrin-gum arabic (MD/GA), and maltodextrin-gum arabic-sodium caseinate (MD/GA/SC) to simulated digestion media.

In the first 90 min, the beetroot extract (85.08%) showed higher radical scavenging activity than all microcapsules (49.46–84.09%), while MD/GA/SC microcapsules had the highest antioxidant activity in the following periods. At the end of the test period (180 min), the radical scavenging activity was determined as MD/GA/SC > beetroot extract >MD/GA > GA > MD from high to low. The radical scavenging activity of the core material occurs in two different mechanisms.^39^ The core material interacts with the DPPH radical in the microcapsule, or the released core material from the microcapsule passes into the test environment and interacts with the DPPH radical. In both cases, the core material must pass the diffusion resistance to pass through the capsule structure and come out. Depending on the length of the transition period, the radical scavenging activity of the central material in the encapsulated samples continues for a longer time compared to the free samples. Samples in the microcapsule structure exhibit higher inhibition activity over a longer period as a result of time-dependent interaction. When the release profile and radical scavenging activity test results were evaluated together, it was determined that both the highest anthocyanin release rate and the highest radical scavenging power were provided by the MD/GA/SC microcapsule. The MD/GA/SC formulation produced a markedly higher encapsulation efficiency and thermal resistance compared with formulations commonly reported in earlier studies, indicating a synergistic stabilizing effect that has not been documented previously. Ersus and Yurdagel^40^ reported that antioxidant activity was highly correlated with the amount of anthocyanins that is consistent with results in this study.

### 3.4 Static In Vitro Digestion

Anthocyanin release from MD, GA, MD/GA, and MD/GA/SC microcapsules is given in Figure 3B. In all microcapsules, anthocyanin release occurred in two stages, fast and slow. The fast-release phase was completed in the first 60 min of the release test with the presence of amylase and pepsin enzymes (pH 3). The amount of anthocyanin released from MD, GA, MD/GA, and MD/GA/SC microcapsules at this stage was determined to be 72.24%, 73.31%, 71.00%, and 69.68%, respectively. The easy release of anthocyanin on the microcapsule surface occurred in the fast phase. In the slow phase period (defined as the plateau period), a longer period was needed for the release of anthocyanin from the inside of microcapsule.

Besides the location of the core in the microcapsule, there were also different parameters such as the wall material hydration or effect of digestion enzymes that may trigger the easy release in the first stage. Ozdemir et al.^41^ reported that the polymer chain was relaxed due to hydrated wall materials in the water-based release medium causing negative bonding to the new structure of wall physically and chemically. In other studies, microencapsulated squalene by using MD, wheat protein, and GA as the wall material determined that digestive enzymes (amylase, protease) trigger the release rate.^42^

At the end of the 4 h release test, the highest release rate was determined in the MD/GA/SC formulation containing the lowest amount of MD (37.5%). The other wall formulations had a higher amount of MD ((MD 100%), MD/GA (50%)) than the MD/GA/SC formulation. Medene et al.^43^ reported that as the MD concentration increases in wall material formulations, the release slows down. The lowest release rate was determined in the microcapsule produced with GA alone (64.25%). The physicochemical properties of the wall material affect the core material’s release characteristics. For instance, the lower release rates determined in studies where gums are used as the wall material are explained by the formation of a hard and dense layer in the emulsion formed during microencapsulation.^44^

### 3.5 Thermal Degradation Kinetics During Heat Treatment

The thermal stabilities of free and microencapsulated beetroot anthocyanins followed by kinetic parameters (*k, t*_1/2_ and *E*_a_) at different temperatures (40, 60, 80, and 100 °C) and pH (2.5, 4.5, and 6.5) are given in Table 2. The anthocyanin degradation rate increased in all samples as the temperature increased. The fastest degradation was observed at pH = 2.5 (Figure 4). The *k* values of free beetroot extract and MD, GA, MD/GA, and MD/GA/SC microcapsules were determined to be 0.056, 0.054, 0.050, 0.014, and 0.013 ppm, respectively, under the most sensitive conditions (100 °C and pH 2.5). Under the same conditions, the half-life (*t*_1/2_) of anthocyanin degradation was increased by 1.09, 1.44, 2.28, and 3.24-fold as a result of microencapsulation with MD, GA, MD/GA, and MD/GA/SC. This result is in accordance with that of Tao et al.^11^ who microencapsulated the blueberry anthocyanin extract by using different combinations of MD, GA, β-cyclodextrin, and whey protein isolate as the wall material. The MD/GA/SC capsules displayed the lowest *k* values under all pH-temperature combinations, indicating superior resistance to thermal and chemical degradation. At extreme conditions (pH 2.5, 100 °C), the ternary matrix preserved anthocyanins more effectively than other formulations. Mechanistically, this enhanced stability can be attributed to the formation of a compact protein-polysaccharide network that limits oxygen diffusion, restricts water mobility, and reduces the extent of pigment exposure to degradation catalysts. This behavior aligns with previous encapsulation studies. For instance, Mazuco et al.^45^ reported only moderate heat protection for juçara anthocyanins encapsulated in MD/GA freeze-dried powders, whereas the present study demonstrated a greater reduction in k values, reflecting stronger thermal protection. Additionally, the pH strongly influenced pigment stability. Anthocyanins remained most stable at pH 2.5 due to the dominance of the flavylium cations, while higher pH values promoted the formation of unstable quinonoidal bases and chalcones, resulting in an accelerated degradation. This pH-dependent behavior matches the classic degradation mechanism reported in the anthocyanin chemistry literature.

**Table 2:** Degradation Kinetics and Arrhenius Parameters of Beetroot Extract and Beetroot Extract-Loaded in Microcapsules of Maltodextrin (MD), Gum Arabic (GA), Maltodextrin/Gum Arabic (MA/GA), and Maltodextrin/Gum Arabic/Sodium Caseinate (MA/GA/SC)

samples	pH	temperature (° C)	degradation kinetic parameters				Arrhenius parameters		
			reaction order	*k* (1/min)	*t*_1/2_ (min)	*R* ^2^	*E*a (kJ/mol)	*R* ^2^	k0 (1/min)
beetroot	2.5	40	first-order	0.018	40.057	0.980	16.634	0.956	11.308
		60	first-order	0.029	37.363	0.976			
		80	first-order	0.034	15.894	0.946			
		100	first-order	0.056	9.8000	0.789			
	4.5	40	first-order	0.015	46.511	0.988	20.785	0.979	8.716
		60	first-order	0.019	36.345	0.985			
		80	first-order	0.025	19.794	0.939			
		100	first-order	0.028	15.503	0.780			
	6.5	40	first-order	0.012	52.105	0.966	23.273	0.909	8.387
		60	first-order	0.013	31.327	0.953			
		80	first-order	0.022	22.010	0.963			
		100	first-order	0.025	18.668	0.755			
MD	2.5	40	first-order	0.017	40.057	0.976	18.400	0.999	4.932
		60	first-order	0.030	23.491	0.983			
		80	first-order	0.048	14.589	0.845			
		100	first-order	0.054	10.625	0.889			
	4.5	40	first-order	0.012	56.341	0.963	25.972	0.989	3.738
		60	first-order	0.015	50.217	0.971			
		80	first-order	0.017	20.694	0.935			
		100	first-order	0.021	15.931	0.792			
	6.5	40	first-order	0.011	61.327	0.903	27.586	0.987	3.300
		60	first-order	0.013	52.500	0.930			
		80	first-order	0.016	22.486	0.976			
		100	first-order	0.019	19.114	0.863			

samples	pH	temperature (° C)	degradation kinetic parameters				Arrhenius parameters		
			reaction order	k (1/min)	*t*_1/2_ (min)	*R* ^2^	*E*_a_ (kJ/mol)	*R* ^2^	k0 (1/min)
GA	2.5	40	first-order	0.017	66.634	0.954	20.134	0.952	4.468
		60	first-order	0.030	53.720	0.974			
		80	first-order	0.048	24.407	0.864			
		100	first-order	0.050	14.142	0.892			
	4.5	40	first-order	0.012	99.000	0.974	24.167	0.998	4.463
		60	first-order	0.015	66.000	0.957			
		80	first-order	0.017	29.117	0.932			
		100	first-order	0.021	18.268	0.822			
	6.5	40	first-order	0.011	110.000	0.915	26.053	0.981	3.027
		60	first-order	0.013	67.281	0.964			
		80	first-order	0.016	32.870	0.978			
		100	first-order	0.019	20.195	0.934			
MD/GA	2.5	40	first-order	0.009	68.166	0.956	33.416	0.980	4.654
		60	first-order	0.010	54.766	0.973			
		80	first-order	0.013	43.100	0.935			
		100	first-order	0.014	22.397	0.770			
	4.5	40	first-order	0.007	103.432	0.994	48.743	0.991	3.469
		60	first-order	0.009	92.400	0.981			
		80	first-order	0.012	45.018	0.949			
		100	first-order	0.014	31.195	0.884			
	6.5	40	first-order	0.005	144.375	0.959	57.027	0.935	2.228
		60	first-order	0.013	67.281	0.964			
		80	first-order	0.016	32.870	0.978			
		100	first-order	0.019	20.195	0.934			
MD/GA/SC	2.5	40	first-order	0.009	103.432	0.979	37.460	0.994	2.886
		60	first-order	0.010	82.500	0.929			
		80	first-order	0.013	59.152	0.954			
		100	first-order	0.013	31.846	0.972			
	4.5	40	first-order	0.007	238.965	0.955	52.517	0.947	2.083
		60	first-order	0.009	128.333	0.953			
		80	first-order	0.012	59.230	0.978			
		100	first-order	0.014	45.468	0.959			
	6.5	40	first-order	0.005	266.538	0.954	62.045	0.947	1.308
		60	first-order	0.013	67.281	0.964			
		80	first-order	0.016	32.870	0.978			
		100	first-order	0.019	20.195	0.934			

**Figure 4:**
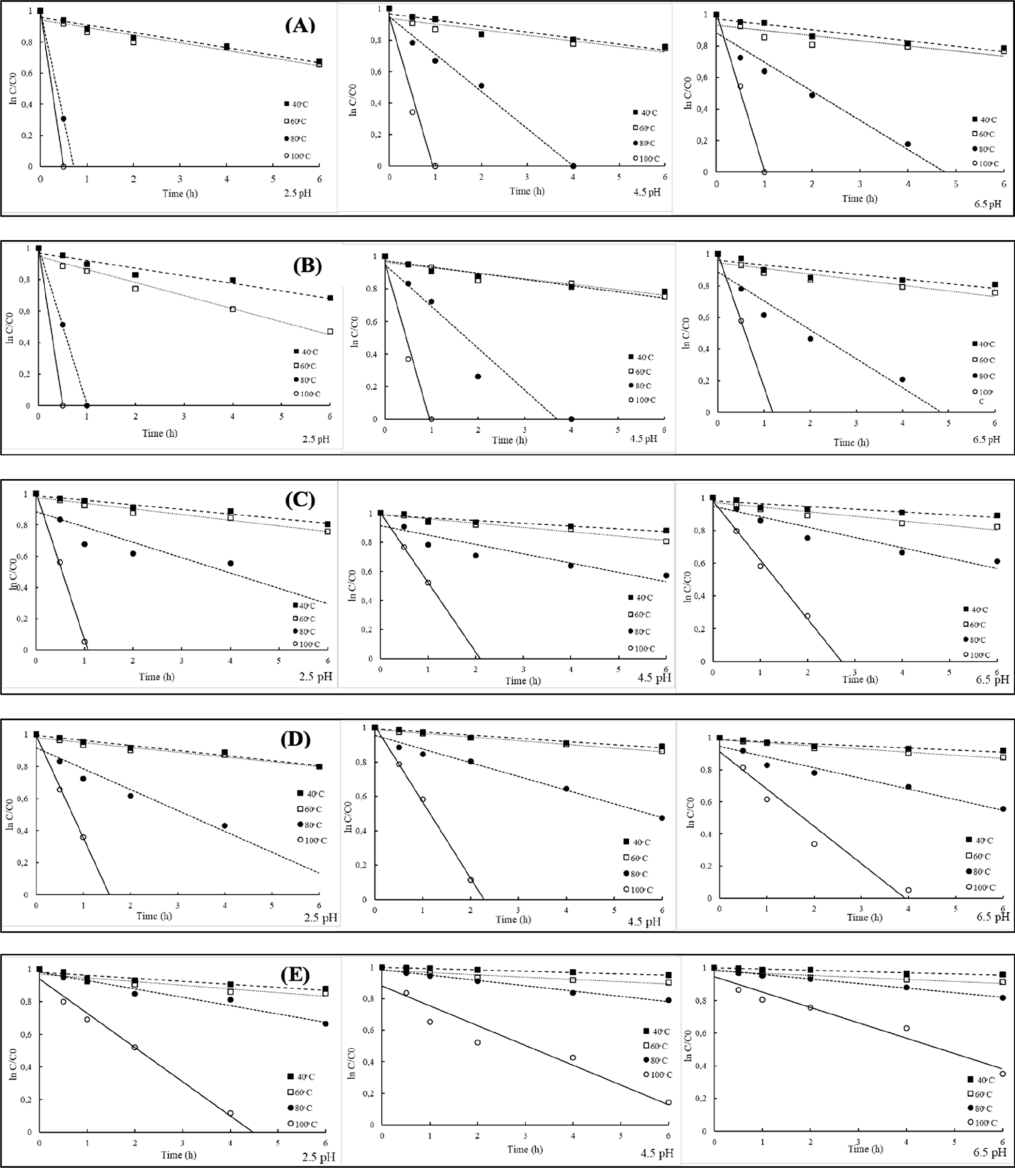
Degradation of anthocyanins of sample during heating at 40, 60, 80, and 100 ° C. (A) beetroot extract, beetroot-loaded microcapsules; (B) maltodextrin (MD), (C) gum arabic (GA), (D) maltodextrin: gum arabic (MA/GA), and (E) maltodextrin/gum arabic/sodium caseinate (MA/GA/SC).

In this study, the *k* value (4.9 × 10^–3^, 6.8 × 10^–3^ 1/min) decreased in all formulations of microencapsules compared to the free sample (7.7 × 10^–3^ 1/min), while the *t*_1/2_ value increased in microencapsulated samples (101.9–141.5 min) compared to the free sample (90.0 min). Another study reported that the microencapsulation of beetroot extract with MD and xanthan gum increased the *t*_1/2_ value from 5.4 days to 6.3 days.^12^

The degree of reaction was decided by comparing the *R* ^2^ values. The anthocyanin degradation in free and microencapsulated beetroot extract followed a first-order reaction pattern (*R* ^2^ range values 0.780–0.988 for free and 0.792–0.976, 0.822–0.978, 0.770–0.988, and 0.922–0.988, MD, GA, MD/GA, and MD/GA/SC for microcapsules, respectively). These results are consistent with the literature on anthocyanin degradation kinetic model results of different fruits. The degradation kinetics of anthocyanins in blackberry^46^ and grape^47^ fit the first-order reaction kinetics.

As can be seen in Table 2, the activation energy (*E*_a_) increased as the pH increased in all of the samples. In order to show the effect of microencapsulation on thermal stability at different pH values, Arrhenius curves were obtained with 1/*T* (1/*K*) versus Ln *k* data (Figure 5). Different increases in *E*_a_ values were observed, depending on the wall materials. The highest *E*_a_ value (37.460 kJ/mol) was determined in the MD-GA-SC mixture followed by MD/GA (33.416 kJ/mol), GA (20.134 kJ/mol), MD (18.400 kJ/mol), and free beetroot extract (16,634 kJ/mol). High *E*_a_ means that anthocyanin molecules need higher energy to collide with each other and initiate the decomposition reaction, while low activation energy means that anthocyanins need very low energy for the decomposition reaction to take place. The thermal stability of anthocyanins increases as the activation energy increases.^49^ In the study by Idham et al.,^48^ roselle anthocyanins were microencapsulated with MD, GA, and soluble starch, and their thermal stability was evaluated. Consistent with our study results, the researchers assessed that microencapsulation enhanced the thermal stability of roselle anthocyanins, as it determined a higher *E*_a_ value in the MD-GA microcapsule (81.09 kJ/mol) compared with the free extract (68.67 kJ/mol). Compared with earlier studies, where *E*_a_ values were generally lower for MD- or GA-based single systems, the ternary matrix in the present work provided a more robust barrier. The enhanced E_a_ can be mechanistically explained by the reduced mobility of reactive species within the protein-polysaccharide network, which increases the energetic threshold needed for degradation reactions.

**Figure 5:**
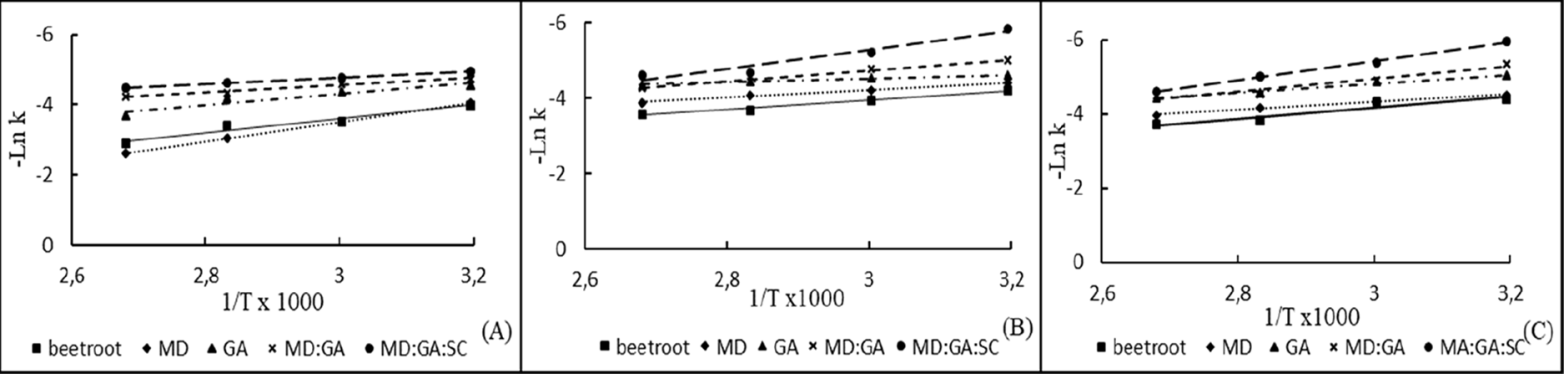
Arrhenius pots for degradation of anthocyanins in different pH during heating, (A) 2.5 pH; (B) 4.5 pH; and (C) 6.5 pH.

The release profile of anthocyanins varied significantly among the formulations. MD alone showed rapid release due to its high solubility and fast hydration, whereas GA provided moderate control because of its denser matrix. The MD/GA/SC combination produced the slowest and most controlled release behavior. This release mechanism is consistent with the expected role of sodium caseinate, which provides hydrophobic interactions and film-forming capacity, both of which slow diffusion of encapsulated pigments. Similar slow-release behavior has been reported for protein-containing encapsulation systems, such as whey protein isolate (WPI)-based microcapsules used for essential oil stabilization^49^.

### 3.6 ANN Model Computation

Calculations made using traditional modeling techniques in the literature show that the anthocyanin degradation fits partially to the first-order reaction pattern. However, this model assumes that the degradation rate has only a first-order dependence that it depends only on the concentration (ignoring variables such as temperature and pH) and that the reaction occurs in a single step and does not involve more than one intermediate step. These assumptions limit the effectiveness of the created model. Generally, real systems often go beyond these simple assumptions and require more complex models.^50,51^ Various parameters, such as coating material-substrate interactions, pore structure, pH, temperature, light, oxygen, enzymes, and solvent types, play an active role in the anthocyanin release rate and degradation process. This can be understood from the *R* ^2^ values of the first-order kinetic model created. The ANN model was able to define the relationship between input parameters and output parameters quickly, practically, and with high harmony, taking into account all conditions and variables, instead of producing separate expressions for each condition as in the first-order kinetic model. This ANN model, created using artificial intelligence techniques, has paved the way for more efficient use of anthocyanins in industrial applications, thanks to the optimization of the shell material composition. The degradation of anthocyanins released from free and microencapsulated beetroot extract under various pH and temperature conditions is nonlinear and complex depending on many parameters. In the developed ANN model, the remaining anthocyanin concentration in the beetroot structure was estimated. The comparison of experimental data and the ANN model predictions are presented in Figure 6.

**Figure 6:**
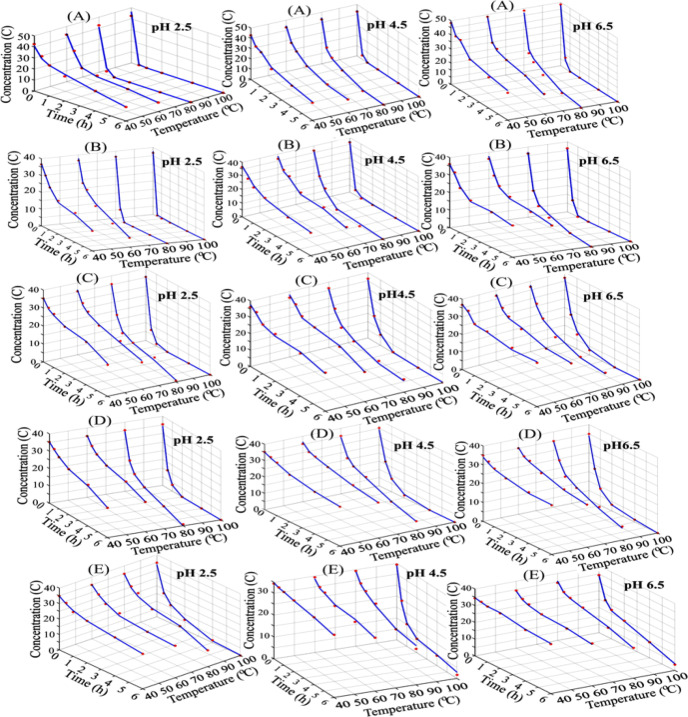
Comparison between the predictions of ANN model and experimental degradation values of the anthocyanin at different pH and temperatures (A) beetroot extract, beetroot extract-loaded microcapsules; (B) maltodextrin (MD), (C) gum arabic (GA), (D) maltodextrin/gum arabic (MA/GA), and (E) maltodextrin/gum arabic: sodium caseinate (MA/GA/SC); blue lines: model predict, red points: experimental values).

The anthocyanin concentrations had a high correlation with the developed ANN model. R^2^, MSE, RMSE and MAPE (%) values to evaluate the success of the ANN model in modeling the anthocyanin degradation behaviors are presented in Table 3. A higher value of the correlation coefficient (*R* ^2^) and smaller values of MSE, RMSE, and MAPE indicate the high performance of the model. Although the degradation of anthocyanin complies with the first-order reaction kinetics as previously reported,^46,48^ no agreement is concluded about the high statistical parameters. Based on experimental data in this study, *R* ^2^ values ranged from 0.770 to 0.988 according to a first-order reaction model of anthocyanin degradation in free and microencapsulated beetroot extracts. The ANN model demonstrated superior predictive performance (*R* ^2^ > 0.98) compared with first-order kinetics used in past research, highlighting the methodological advantage of incorporating artificial intelligence for degradation modeling.

**Table 3:** Performance Indices Achieved by Using ANN during Test Periods^a^

parameters	beetroot	MD	GA	MD/GA	MD/GA/SC
RMSE	2.2112	2.3933	2.0577	1.6202	1.3161
MSE	4.8896	5.7277	4.2339	2.6251	1.7321
MAPE (%)	5.4156	7.1852	6.6783	4.1285	7.5813
*R*2	0.9894	0.9887	0.9821	0.9917	0.9813

a RMSE: Root mean square error, MSE: mean square error, MAPE: mean absolute percentage error, *R* ^2^: coefficient of determination, MD: maltodextrin, GA: Gum arabic, SC: sodium caseinate.

The ANN model was able to define the relationship between input parameters and output parameters quickly, practically and with high harmony, taking into account all conditions and variables, instead of producing separate expressions for each condition as in the first-order kinetic model. The comparison of the results of this study with the recent literature is presented in Table 4. This ANN model, created using an artificial intelligence technique, has paved the way for more efficient use of anthocyanins in industrial applications, thanks to the optimization of the shell material composition.

**Table 4:** Comparison of Present Study with the Recent Literature

parameter	beetroot anthocyanin, MD/GA/SC (this study)	barberry anthocyanin,^52^ MD/GA	Juçara anthocyanin,^45^ MD/GA	purple corn anthocyanin,^49^ MD/GA/WPI
encapsulation efficiency (EE)	93.36%	92.8%	74–89%	80–90%
thermal stability	lowest degradation rate constants across all pH–temperature combinations	moderate stability	degradation increases with temperature	moderate improvement
activation energy (*E*_a_)	highest *E*_a_	not reported	moderate	moderate
release behavior	controlled and slow release	generally fast release	moderate	formulation-dependent
modeling approach	ANN (*R* ^2^ > 0.98)	not used	not used	not used
application potential	high potential in heat-processed foods and natural colorants	limited at high temperature	suitable for freeze-dried products	limited direct food applications

The MD/GA/SC formulation demonstrated superior thermal resistance, exhibiting the lowest degradation rate constants across all of the pH-temperature combinations tested. When compared with previously reported freeze-dried anthocyanin systems, such as the juçara anthocyanin microcapsules described by Mazuco et al.^45^ the markedly reduced k values observed in this study indicate a stronger protective capacity of the ternary wall matrix, particularly under harsh conditions (pH 2.5, 100 °C). This enhanced stability is further supported by the substantially higher activation energy obtained for the MD/GA/SC system, suggesting the formation of a more robust diffusion and protection barrier, relative to earlier encapsulation approaches. Moreover, the controlled and sustained release behavior of the MD/GA/SC microcapsules contrast with the more rapid release profiles typically reported for single-component MD or GA systems,^52^ highlighting the functional advantages of the ternary matrix in regulating anthocyanin delivery. Finally, unlike previous studies in which degradation behavior was modeled exclusively through conventional kinetic approaches, the incorporation of an artificial neural network (ANN) in this work provided exceptional predictive performance (*R* ^2^ > 0.98), effectively capturing the nonlinear and multivariate degradation patterns that first-order models fail to represent.

The findings obtained in this study indicate strong application potential in various food and nutraceutical systems. The enhanced thermal and pH stability provided by the MD/GA/SC microencapsulation matrix makes this approach highly suitable for heat-processed foods, beverages, dairy products, and confectionery items, in which natural pigment stability is essential. The controlled release behavior observed also suggests that this encapsulation strategy may be utilized in nutraceutical formulations requiring a targeted or sustained release of bioactive compounds. Furthermore, the successful implementation of ANN modeling offers a practical tool for industrial formulation optimization, enabling the rapid prediction of anthocyanin degradation under different processing conditions and supporting data-driven product development.

Although this study helps clarify how the MD/GA/SC matrix improves both the stability and the release behavior of beetroot anthocyanins, there are still several points that deserve attention in future research. For example, it would be useful to see how these microcapsules perform once they are mixed into real food systems, where proteins, sugars, fats, and other ingredients might influence their release or breakdown in ways that are not fully represented in controlled laboratory solutions. Longer storage trials under different environmental conditions, as well as experiments that simulate the digestion process, could also provide more realistic information about how well the encapsulated pigments hold up and how accessible they remain in actual consumption scenarios. Testing whether these capsules can be manufactured at a larger scale, for instance, using spray-drying or fluidized-bed methods, would also help determine whether the system is suitable for industrial applications. In addition, the ANN model developed here could be expanded to predict more than one output at a time or adapted to different biopolymer systems, which may open the door to more sophisticated and data-oriented encapsulation design strategies.

## 4 Conclusion

In this study, several wall material systems, MD, GA, their combined form, and the more complex MD/GA/SC mixture, were examined to see how well they could protect beetroot anthocyanins once encapsulated. Among all of these options, the ternary formulation stood out and consistently delivered the strongest performance. It achieved a higher encapsulation efficiency, preserved antioxidant activity more effectively, and showed a slower more controlled release compared with that of the other matrices. These results suggest that the structural network formed by MD, GA, and SC provides a more secure environment for the pigments. The stability tests conducted across different pH and temperature conditions reinforced this observation. The MD/GA/SC capsules exhibited lower degradation rate constants and longer half-life values, even under harsh settings, such as pH 2.5 and 100 °C. The noticeable rise in activation energy also supports the idea that this ternary structure offers a stronger barrier against heat-induced breakdown, which fits well with what is known about the protective roles of combined polysaccharide-protein matrices. A notable feature of this work is the integration of an ANN model to characterize anthocyanin degradation. Compared with the traditional first-order kinetic approach, the ANN provided more accurate predictions, likely because it can handle interactions that are nonlinear and influenced by several variables at once. This suggests that ANN-based tools could be quite useful for refining encapsulation designs and for forecasting the behavior of sensitive bioactives in more complex situations. Overall, the outcomes indicate that MD/GA/SC is a promising and practical wall material for improving anthocyanin stability and may hold value for wider industrial applications. The successful use of ANN modeling also points toward a methodological direction that could become increasingly important as food and nutraceutical research moves further toward data-driven approaches.
